# Novel Scorpion Toxin ω-Buthitoxin-Hf1a Selectively Inhibits Calcium Influx via Ca_V_3.3 and Ca_V_3.2 and Alleviates Allodynia in a Mouse Model of Acute Postsurgical Pain

**DOI:** 10.3390/ijms25094745

**Published:** 2024-04-26

**Authors:** Dan Wang, Volker Herzig, Zoltan Dekan, K. Johan Rosengren, Colton D. Payne, Md. Mahadhi Hasan, Jiajie Zhuang, Emmanuel Bourinet, Lotten Ragnarsson, Paul F. Alewood, Richard J. Lewis

**Affiliations:** 1 Department of Chinese Medicine and Pharmacy, School of Pharmacy, Jiangsu University, Zhenjiang 212013, China; 3201603097@stmail.ujs.edu.cn; 2 Division of Chemistry and Structural Biology, Institute for Molecular Bioscience, The University of Queensland, Brisbane, QLD 4072, Australial.ragnarsson@imb.uq.edu.au (L.R.); p.alewood@imb.uq.edu.au (P.F.A.); 3 Centre for Bioinnovation, University of the Sunshine Coast, Sippy Downs, QLD 4556, Australia; vherzig@usc.edu.au; 4 School of Science, Technology and Engineering, University of the Sunshine Coast, Sippy Downs, QLD 4556, Australia; 5 School of Biomedical Sciences, The University of Queensland, Brisbane, QLD 4072, Australia; j.rosengren@uq.edu.au (K.J.R.); c.payne@uq.net.au (C.D.P.); 6 Pharmacy Discipline, Life Science School, Khulna University, Khulna 9208, Bangladesh; mahadhi@pharm.ku.ac.bd; 7 Institute of Functional Genomics, Montpellier University, CNRS, INSERM, 34090 Montpellier, France; emmanuel.bourinet@igf.cnrs.fr

**Keywords:** venom peptides, T-type calcium channels, peripheral pain, acute post-surgical pain

## Abstract

Venom peptides have evolved to target a wide range of membrane proteins through diverse mechanisms of action and structures, providing promising therapeutic leads for diseases, including pain, epilepsy, and cancer, as well as unique probes of ion channel structure-function. In this work, a high-throughput FLIPR window current screening assay on T-type Ca_V_3.2 guided the isolation of a novel peptide named ω-Buthitoxin-Hf1a from scorpion *Hottentotta franzwerneri* crude venom. At only 10 amino acid residues with one disulfide bond, it is not only the smallest venom peptide known to target T-type Ca_V_s but also the smallest structured scorpion venom peptide yet discovered. Synthetic Hf1a peptides were prepared with C-terminal amidation (Hf1a-NH_2_) or a free C-terminus (Hf1a-OH). Electrophysiological characterization revealed Hf1a-NH_2_ to be a concentration-dependent partial inhibitor of Ca_V_3.2 (IC_50_ = 1.18 μM) and Ca_V_3.3 (IC_50_ = 0.49 μM) depolarized currents but was ineffective at Ca_V_3.1. Hf1a-OH did not show activity against any of the three T-type subtypes. Additionally, neither form showed activity against N-type Ca_V_2.2 or L-type calcium channels. The three-dimensional structure of Hf1a-NH_2_ was determined using NMR spectroscopy and used in docking studies to predict its binding site at Ca_V_3.2 and Ca_V_3.3. As both Ca_V_3.2 and Ca_V_3.3 have been implicated in peripheral pain signaling, the analgesic potential of Hf1a-NH_2_ was explored in vivo in a mouse model of incision-induced acute post-surgical pain. Consistent with this role, Hf1a-NH_2_ produced antiallodynia in both mechanical and thermal pain.

## 1. Introduction

Scorpions are venomous arachnids of the order Scorpiones, with around 2798 currently (December 2023) described species distributed on all continents except Antarctica [1]. Scorpion venoms are complex mixtures of peptides, enzymes, mucoproteins, amino acids (aa), nucleotides, lipids, amines, heterocyclic components, inorganic salts, and probably other yet to be described substances [2]. They are rich sources of neurotoxic polypeptides that recognize and bind to ion channels of excitable and non-excitable cells and modulate their functional properties. Based on the specific binding properties, scorpion toxins are divided into sodium channel, potassium channel, chloride channel, and calcium channel (Ca_V_) toxins [3] and further clustered into 26 subfamilies according to structure, mechanism of action, and binding site on different channel subtypes [4,5]. The sodium channel-specific long-chain toxins (60–76 aa residues), subdivided into α- and β-toxins targeting receptor sites 3 and 4 of vertebrate sodium channels, are the most numerous and well-studied scorpion toxins [6,7,8]. Calcium channel toxins are less studied, with the 63-aa kurtoxin from venom of the scorpion *Parabuthus transvaalicus* being the first and only scorpion toxin shown to selectively bind T-type Ca_V_s (Ca_V_3.1 and Ca_V_3.2) [9,10].

Low voltage-activated (LVA) T-type Ca_V_s are key players in signal amplification and synaptic integration, and a privileged gate for calcium influx that initiates many physiological events like secretion, neurotransmission, and cell proliferation. A high density of T-type Ca_V_s are expressed in sensory neurons [11,12,13], and many in vitro and in vivo studies have demonstrated a significant role for T-type Ca_V_3.2 [14,15,16,17,18] in both peripheral nociception and central sensitization, and for Ca_V_3.3 [19,20] in peripheral neuropathy. Sensitization of sensory neurons contributes to the appearance of allodynia and hyperalgesia, which allows pain responses from normally innocuous stimuli (allodynia) or amplified and heightened pain responses from noxious stimuli (hyperalgesia) [21]. Central sensitization can cause long-term changes in pain pathways and increase the difficulty of treating pain. As a major debilitating symptom of many diseases and a disease itself if persisting beyond recovery from illness, pain has been reported to be a leading cause of disability in countries like the US [22]; however, it remains undermanaged. Post-surgical pain, as a typical example, has a high incidence in many types of surgery [23], but the need for efficient pain management to prevent the development of chronic pain and other negative effects on patient recovery is largely unmet [24]. There are many ongoing studies on the development of analgesics with better efficacy and safety. Several voltage-gated ion channels contributing to the sensitization of sensory neurons, including sodium channels Na_V_1.1 [25] and Na_V_1.7 [26] and high voltage-activated (HVA) N-type Ca_V_2.2 [27] and LVA T-type Ca_V_3.2 [13,15,28,29], have emerged as potential targets for new analgesic therapies. Venom peptides that selectively target pain-related ion channels provide novel sources of new analgesic leads with the potential to generate fewer side effects.

In this work, a high-throughput FLIPR window current screening assay for Ca_V_3.2 was used to guide crude venom fractionation. T-type window currents typically operate between −80 and −30 mV [30] and could be eliminated by hyperpolarization [31]. The Ca_V_3.2 alone cell line used in our screening assay has been proven to have optimal membrane potential for window currents [31]. Screening of venom fractions identified one scorpion, *Hottentotta franzwerneri* fraction (*t*_R_ = 13.5 min), that showed 66% inhibition of Ca_V_3.2 window current at 48 µM. N-terminal sequencing of the fraction resulted in a major sequence of HCTPNSNHCW, which was named ω-Buthitoxin-Hf1a, following rational nomenclature for naming peptide toxins [32]. Synthetic C-terminally amidated (Hf1a-NH_2_) and free C-terminus form (Hf1a-OH) of the peptide were made by chemical synthesis. Further characterizations revealed Hf1a-NH_2_, a partial and selective inhibitor of Ca_V_3.2 and Ca_V_3.3 depolarized currents in vitro, and it showed analgesic effects in vivo in a mouse model of acute post-surgical pain.

## 2. Results

### 2.1. High-Throughput FLIPR Venom Screening at Ca_V_3.2

**Figure 1:**
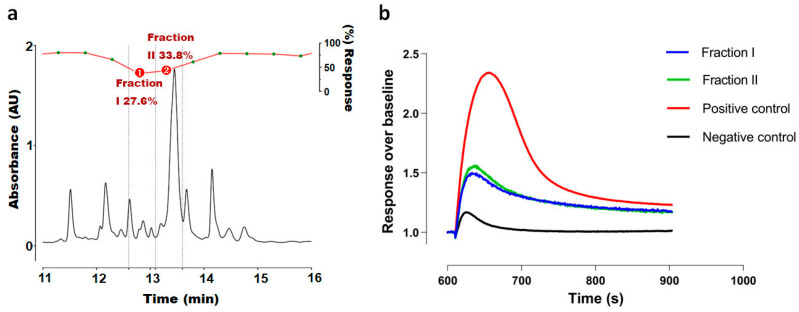
(**a**) RP-HPLC trace of *H. franzwerneri* fractions (indicated in green points) I and II and their maximum responses at Ca_V_3.2 in the FLIPR assay; (**b**) Response over baseline traces of the two fractions I and II isolated from scorpion *H. franzwerneri*, showing 72.4% and 66.2% inhibition of Ca^2+^ responses in Ca_V_3.2, respectively.

A scorpion venom library containing 55 crude venoms from 52 scorpion species of 20 genera across 6 families of scorpions was screened in the FLIPR window current screening assay for Ca_V_3.2 (summarized in Supplementary Materials Figure S1). The Ca_V_3.2 window current screening assay was established to provide a wider net for the initial screening of active compounds, as the current size could be affected by both activation and inactivation modulators. Inhibitory crudes were selected for HPLC fractionation according to potency (>50% inhibition, Table S1) and venom availability, and further screening of the venom fractions led to the identification of active fractions I and II from the scorpion *H. franzwerneri* (Figure 1).

### 2.2. Determination of Peptide Sequence and NMR Structure

Active fraction II showed a dominant mass of 1196.4 Da, and fraction I showed a dominant mass of 1195.4 Da (Figure S2), suggesting that they might correspond to the C-terminal acid and amidated form of the same peptide. Fraction II was purified to homogeneity and sent for N-terminal sequencing, which resulted in a major sequence of HCTPNSNHC(W), which we named ω-Buthitoxin-Hf1a. The C-terminal Trp (W) sequencing signal was weak, but the calculated masses for the free acid (1196.4 Da) and amidated (1195.4 Da) forms supported the observed masses of fractions II and I, respectively. The sequence was analyzed in NCBI Protein Blast, and the results revealed that ω-Buthitoxin-Hf1a is a novel peptide with no significant similarity (<40%) to reported peptides of similar sequence length. To be more precise, the sequence was further subjected to blast in Arachnoserver [33], which also showed ≤62% identities with ≥51 aa spider peptides, scored 18.5 bits, indicating it with no ancestral forms being identified. The best matches were with the µ-TRTX-Hhn2 toxins (62% identity), including µ-TRTX-Hhn2f, µ-TRTX-Hhn2l, µ-TRTX-Hhn2m, µ-TRTX-Hhn2d, µ-TRTX-Hhn2e, µ-TRTX-Hhn2q, and µ-TRTX-Hhn2I. These spider toxins shared a common sequence of CTPGKNEC and included the conserved sequence CTP– –N–C found in Hf1a.

**Figure 2:**
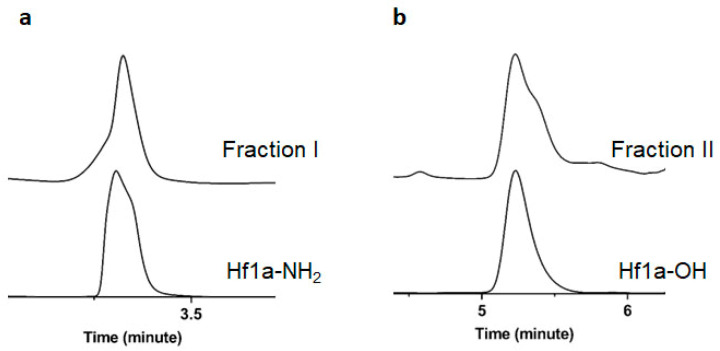
Co-elution of the native and synthetic peptides. (**a**) Co-elution of native fraction I with synthetic Hf1a-NH_2_. (**b**) Co-elution of native fraction II with synthetic Hf1a-OH.

Hf1a was produced as a C-terminal amide (Hf1a-NH_2_) and as a C-terminal acid (Hf1a-OH) by solid-phase peptide synthesis. After preparative chromatography, its purity (>98%) was confirmed by analytical RP-HPLC and correct mass confirmed by LC-MS (Figure S3). As expected, Hf1a-NH_2_ co-eluted well with the main component in fraction I, whereas Hf1a-OH co-eluted well with the main component in fraction II (Figure 2).

**Figure 3:**
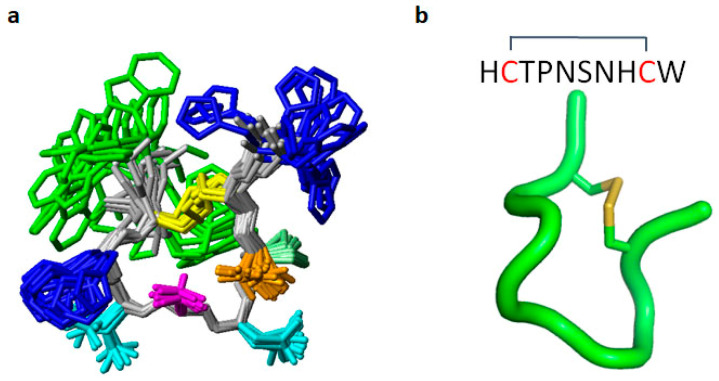
The NMR structure of the C-terminally amidated ω-Buthitoxin-Hf1a. (**a**) Superposition of the 20 lowest energy structures of ω-Buthitoxin-Hf1a, with cysteine shown in yellow, histidine in blue, asparagine in cyan, threonine in orange, proline in light green, serine in pink, and tryptophane in green. (**b**) The lowest energy NMR solution structure of the ω-Buthitoxin-Hf1a backbone.

Hf1a-NH_2_ was then subjected to solution NMR spectroscopy studies. It produced data of high quality, allowing complete resonance assignment of all backbone and side chain ^1^H, ^13^C, and ^15^N nuclei. A full 3D structure was calculated using simulated annealing based on the distance and dihedral restraints derived from the NMR data. The peptide adopts a highly ordered fold within the disulfide constrained cycle comprising residues 2–9, as evident from a backbone RMSD of 0.43 Å, and the structural quality was within the 99th percentile according to MOLPROBITY analysis (Figure 3; statistics table shown in Supplementary Materials Table S2). The structure is characterized by two β-turns, comprising residues 3–6 and 6–9, which are stabilized by hydrogen bonds 6HN-3O and 9HN-6O, respectively. Pro4 adopts a trans conformation based on NOE patterns and ^13^C chemical shifts.

### 2.3. Evaluation of LVA and HVA Ca_V_s Activities of the Synthetic ω-Buthitoxin-Hf1a

#### 2.3.1. Electrophysiological Characterization of the Scorpion Peptide ω-Buthitoxin-Hf1a in QPatch Assays

**Figure 4:**
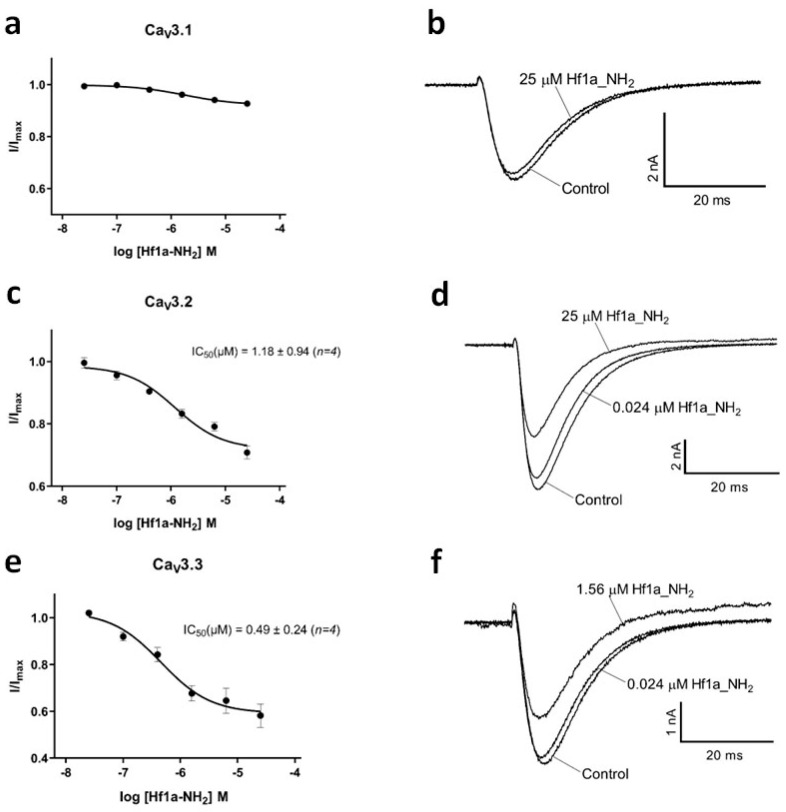
Effect of Hf1a-NH_2_ on Ca_V_3.1, Ca_V_3.2, and Ca_V_3.3 current. (**a**) Hf1a-NH_2_ significantly inhibited (6.6%) the recombinant hCa_V_3.1 channel current at 25 μM (*p* < 0.05; *n =* 4), while lower concentrations were ineffective; (**b**) representative *I*_Ca_ during 200 ms depolarizations to *V*_max_ (−20 mV) from a holding potential of −90 mV before and after perfusions of 25 µM of Hf1a-NH_2_, as indicated. (**c**) Concentration response curve of Hf1a-NH_2_ on recombinant hCa_V_3.2 channels with 31.7% current inhibition at 25 μM (*n =* 4); (**d**) representative *I*_Ca_ during 200 ms depolarizations to *V*_max_ (−25 mV) from a holding potential of −90 mV before and after perfusions of 0.024 µM and 25 µM of Hf1a-NH_2_, as indicated. (**e**) Concentration response curve of Hf1a-NH_2_ on recombinant hCa_V_3.3 channels with 44.6% current inhibition at 25 μM (*n =* 4); (**f**) representative *I*_Ca_ during 200 ms depolarizations to *V*_max_ (−10 mV) from a holding potential of −90 mV before and after perfusions of 0.024 µM and 1.56 µM of Hf1a-NH_2_, as indicated. Data are means ± SEM.

Synthetic Hf1a-NH_2_ and Hf1a-OH were then subjected to electrophysiological characterization, and their effects were examined on human T-type Ca_V_s whole-cell currents in QPatch assays. As shown in Figure 4, Hf1a-NH_2_ showed concentration-dependent partial inhibition of Ca_V_3.2 (IC_50_ = 1.18 ± 0.94 μM, *n* = 4) and Ca_V_3.3 (IC_50_ = 0.49 ± 0.24 μM, *n* = 4) depolarized currents but was ineffective for Ca_V_3.1. The inhibition did not shift the current–voltage (*I*–*V*) curves of Ca_V_3.2 and Ca_V_3.3 (Figure S4). In contrast, Hf1a-OH failed to show activity in any of the three subtypes.

#### 2.3.2. Characterization of ω-Buthitoxin-Hf1a in HVA Calcium Channel FLIPR Assays

**Figure 5:**
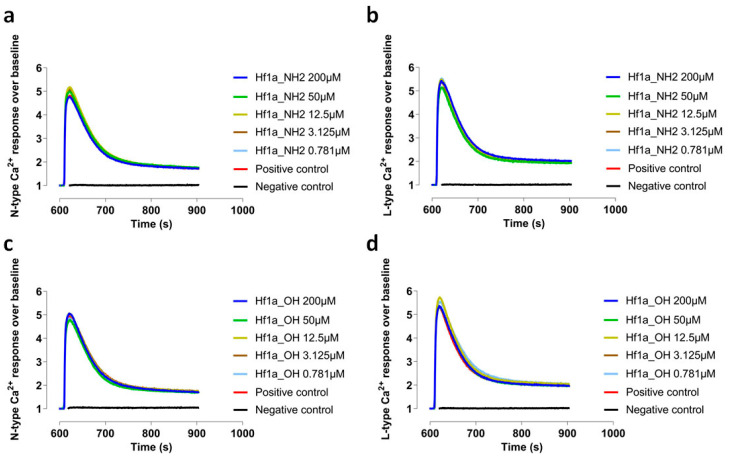
Representative N-type (**a**) and L-type (**b**) Ca^2+^ response over baseline traces with addition of Hf1a_NH_2_ (0.781–200 µM); and N-type (**c**) and L-type (**d**) Ca^2+^ response over baseline traces with addition of Hf1a_OH (0.781–200 µM).

To test the selectivity of ω-Buthitoxin-Hf1a, both synthetic Hf1a-NH_2_ and Hf1a-OH were tested for activity against the human HVA N-type Ca_V_2.2 and L-type Ca_V_s in SH-SY5Y FLIPR assays, but as shown in Figure 5, neither form of ω-Buthitoxin-Hf1a affected N-type or L-type Ca_V_s at 0.781–200 µM (*n* = 4).

#### 2.3.3. ω-Buthitoxin-Hf1a Docking in T-Type Calcium Channels

**Figure 6:**
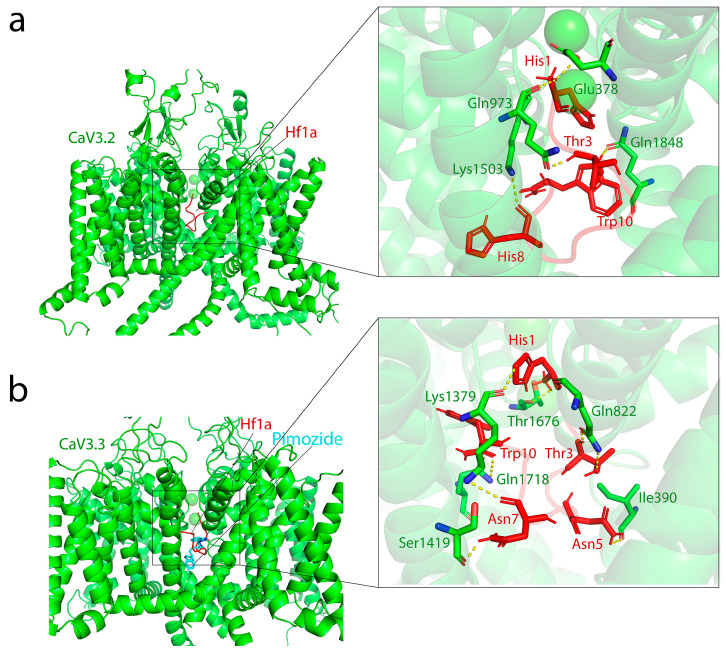
Predicted binding mode of Hf1a (colored red) in human Ca_V_3.2 and Ca_V_3.3 (colored green). (**a**) General view of the lowest energy docking pose of Hf1a binding to the Ca_V_3.2 central cavity of the Ca^2+^ permeation pore under the selectivity filter of the channel; local view highlights the interactions of His1 from Hf1a with Glu378 and Gln973 in Ca_V_3.2, whereas Thr3, His8, and Trp10 showed polar interaction with Gln973, Lys1503, and Gln1848, respectively. (**b**) General view of the lowest energy docking pose of Hf1a binding to the Ca_V_3.3 central cavity of the Ca^2+^ permeation pore under the selectivity filter of the channel, which is the published binding site of pimozide (colored cyan) in Ca_V_3.3; local view highlights the interactions of His1 of Hf1a with Lys1379 and Thr1676 in Ca_V_3.3, and interactions of Asn7 with Ser1419 and Lys1379. Other significant interactions were found between Thr3-Gln822, Asn5-Ile390, and Trp10-Gln1718. Predicted hydrogen bonds are shown as yellow dashed lines with all distances less than 4 Å.

The binding sites of mibefradil and pimozide have been identified in the central cavity of the Ca^2+^ permeation pore, under the selectivity filter of the Ca_V_3.3 channel [34]. Considering that Hf1a is a small peptide and did not shift *I*–*V* curves of Ca_V_3.2 and Ca_V_3.3 in its inhibition, we postulated that it may target the same site and performed molecular docking in the central pore region of Ca_V_3.1, Ca_V_3.2, and Ca_V_3.3. Docking simulations revealed that Hf1a could bind in the central pore region of Ca_V_3.2 (affinity score: −6.4 kcal/mol) and Ca_V_3.3 (affinity score: −5.2 kcal/mol). However, Hf1a failed to dock energy efficiently in the central pore region of Ca_V_3.1 (affinity score: 89.3 kcal/mol) and instead docked preferably above the selectivity filter of the channel (affinity score: −4.8 kcal/mol). According to pairwise interaction analysis, His1, Thr3, and Trp10 from Hf1a are the most important residues, as they are involved in multiple interactions with Ca_V_3.2 and Ca_V_3.3 channel residues (Figure 6). In Ca_V_3.2, His1 formed polar interactions with Glu378 and Gln973, whereas Thr3, His8, and Trp10 showed polar interactions with Gln973, Lys1503, and Gln1848, respectively (Figure 6a). In Ca_V_3.3, His1 formed polar interactions with Lys1379 and Thr1676, and Asn7 formed polar interactions with Ser1419 and Lys1379 (Figure 6b). Other significant interactions were found between Thr3-Gln822, Asn5-Ile390, and Trp10-Gln1718 (Figure 6b).

### 2.4. Local Effects of Hf1a-NH_2_ on Incision-Induced Mechanical and Thermal Allodynia

#### 2.4.1. A mouse Model of Post-Surgical Pain Based on Plantar Incision Leads to Mechanical and Thermal Allodynia

**Figure 7:**
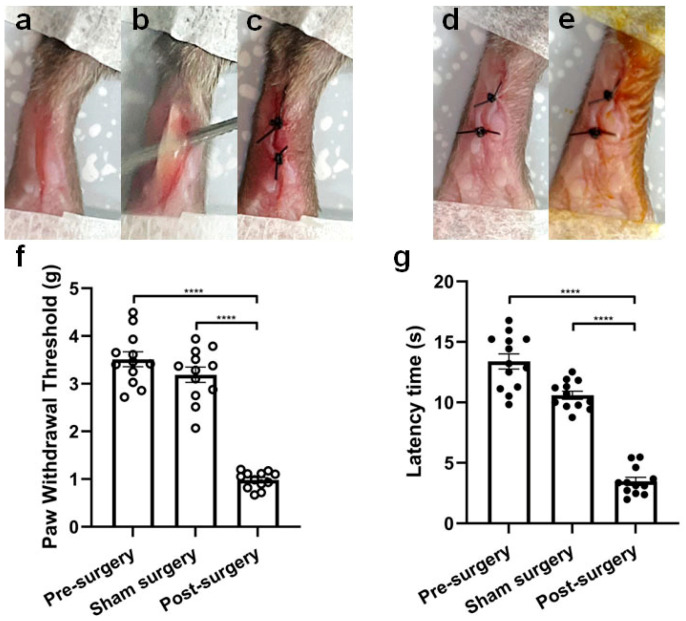
Behavioral characterization of a mouse model of incision-induced acute post-surgical pain. Photographs of crucial steps of incisional surgery (**a**–**c**) and sham surgery (**d**,**e**) on the mouse hind paw. (**a**) A 7-mm longitudinal incision is made through the glabrous skin and fascia of the plantar surface of the right hind paw using a number 11 sterile surgical scalpel. The incision started about 3 mm from the proximal edge of the heel and extended toward the toes. (**b**) The underlying *flexor digitorum brevis* muscle is elevated to mimic muscle retraction and incised longitudinally with its origin and insertion intact. (**c**) The wound was closed with two sterile sutures after hemostasis, using the simple interrupted suture technique. (**d**) Two sterile sutures were carefully stitched into the skin during anesthesia in sham surgery. (**e**) The suturing area was disinfected with 5% povidone–iodine solution. Behavioral tests (mechanical and thermal) to characterize the mouse model of post-surgical pain (**f**,**g**). (**f**) Mechanical allodynia is present 24 h post-surgery with a significantly reduced paw withdrawal threshold in the surgery group compared to the sham group and compared to pre-surgery controls (*n* = 12 per group; **** *p* < 0.0001; one-way ANOVA with Dunnett’s multiple comparisons test). (**g**) Thermal allodynia is present 24 h post-surgery with a significantly reduced paw withdrawal latency time in the surgery group compared to the sham group and compared to pre-surgery controls (*n* = 12 per group; **** *p* < 0.0001; one-way ANOVA with Dunnett’s multiple comparisons test).

The crucial steps of incisional surgery and sham surgery on the mouse right hind paw are shown in Figure 7a–e. The incision significantly (*p* < 0.0001) elicited mechanical allodynia in mice, and a much lower force (0.98 ± 0.05 g) was needed to induce pain when measured 24 h post-surgery compared to sham surgery mice (3.19 ± 0.16 g) and naïve mice (3.51 ± 0.16 g) (Figure 7f). In the previous establishment of the mouse model of post-surgical pain [35], temperature differences to elicit pain were compared among groups, and post-surgery mice showed thermal allodynia with about 2 °C lower paw withdrawal temperature compared to naïve mice (*p* < 0.01); however, this temperature difference was not enough to measure or compare analgesic efficacy of compounds. In this work, we used infrared radiant heat instead, and latency time was measured and compared among groups. As shown in Figure 7g, thermal allodynia was induced by the incision, which significantly (*p* < 0.0001) lowered the latency time of post-surgery mice (3.47 ± 0.33 s) compared to the latency time of naïve mice (13.39 ± 0.62 s) as well as sham surgery mice (10.60 ± 0.34 s).

#### 2.4.2. Hf1a-NH_2_ Effectively Reduces Mechanical and Thermal Allodynia after Surgery

**Figure 8:**
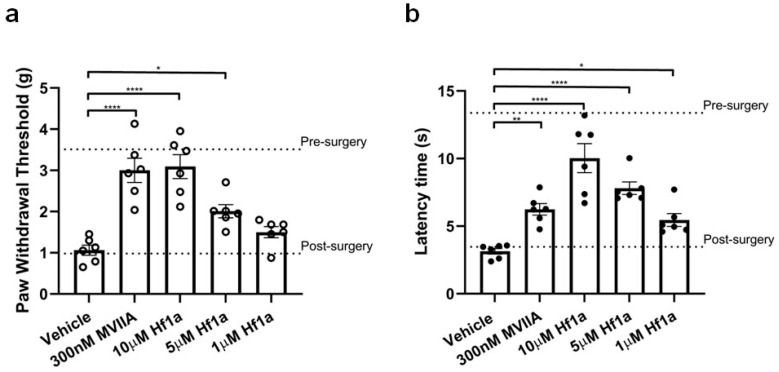
Analgesic effects of Hf1a-NH_2_ on incision-induced mechanical and thermal allodynia, assessed 24 h post-surgery in mice. (**a**) Compared with vehicle control, the paw withdrawal thresholds of post-surgery mice increased significantly after intraplantar injection of 0.1 and 0.2 nmol/paw (5 μM and 10 μM; 20 μL) Hf1a-NH_2_ and 6.0 pmol/paw (300 nM; 20 μL) control peptide MVIIA (* *p* < 0.05; **** *p* < 0.0001; **** *p* < 0.0001, respectively; *n* = 6 per group); (**b**) Compared with vehicle control, the paw withdrawal latency time of post-surgery mice increased significantly after intraplantar injection of 0.02, 0.1, and 0.2 nmol/paw (1 μM, 5 μM and 10 μM; 20 μL) Hf1a-NH_2_ and 6.0 pmol/paw (300 nM; 20 μL) control peptide MVIIA (* *p* < 0.05; **** *p* < 0.0001; **** *p* < 0.0001; ** *p* < 0.01, respectively; *n* = 6 per group); Statistical significance was determined using one-way ANOVA with Dunnett’s multiple comparisons test.

Incision-induced mechanical allodynia in 24 h post-surgery mice was significantly reversed by intraplantar administration of 0.1 and 0.2 nmol/paw (5 μM and 10 μM; 20 μL) Hf1a-NH_2_, which showed significantly higher paw withdrawal thresholds of 2.01 ± 0.16 g (*p* < 0.05) and 3.09 ± 0.29 g (*p* < 0.0001), respectively, compared to the paw withdrawal threshold of vehicle-treated mice (1.06 ± 0.12 g) (Figure 8a). The intraplantar administration of 6.0 pmol/paw (300 nM; 20 μL) control peptide MVIIA also significantly increased the paw withdrawal threshold to 3.00 ± 0.29 g (*p* < 0.0001), which is consistent with a previous report [36]. Surprisingly, the intraplantar administration of 20 pmol/paw (1 μM; 20 μL) Hf1a-NH_2_ significantly increased the paw withdrawal latency time (5.46 ± 0.47 s) of thermal allodynia in 24 h post-surgery mice compared to the latency time (3.14 ± 0.21 s) of vehicle-treated mice (*p* < 0.05) (Figure 8b). The intraplantar administration of 0.1 and 0.2 nmol/paw (5 μM and 10 μM; 20 μL) Hf1a-NH_2_ significantly increased the latency time to 7.80 ± 0.46 s (*p* < 0.0001) and 10.04 ± 1.08 s (*p* < 0.0001), respectively. The intraplantar administration of 6.0 pmol/paw (300 nM; 20 μL) control peptide MVIIA also significantly increased the latency time to 6.24 ± 0.43 s (*p* < 0.01). As assessed 24 h post-injection of the peptides, these peptides showed no adverse effects on locomotor performance of the mice (Figure S5).

## 3. Discussion

A high-throughput FLIPR window current screening assay for Ca_V_3.2 was used to screen our scorpion venom library for inhibitors. We successfully identified a novel, Ca_V_3.2 and Ca_V_3.3 selective scorpion venom peptide with promising analgesic activity in a mouse in vivo acute post-surgical pain assay.

Specifically, a FLIPR window current screening assay for Ca_V_3.2 guided the fractionation of scorpion *H. franzwerneri* crude venom and identified one active fraction (*t*_R_ = 13.5 min) that blocked the Ca_V_3.2 window current at 48 µM (66% inhibition). Derived from this fraction, the active 10 aa peptide HCTPNSNHCW was named ω-Buthitoxin-Hf1a following rational nomenclature for naming peptide toxins [32], and blast results revealed that it is a novel peptide. To our knowledge, ω-Buthitoxin-Hf1a is the smallest venom peptide known to target T-type Ca_V_s and excitingly, the smallest structured peptide containing a disulfide bond isolated from scorpion venoms.

The ≥51 aa µ-TRTX-Hhn2 toxins that best matched our small peptide ω-Buthitoxin-Hf1a in Arachnoserver blast (62% identity) shared a conserved sequence of CTP– –N–C with Hf1a, of which Thr3 was revealed in our docking studies to be one of the key residues in its interaction with T-type Ca_V_s. According to Arachnoserver, these µ-TRTX-Hhn2 toxins have been presumed to have sodium channel inhibitory activity, based on the homology with μ-theraphotoxin-Hhn2a (hainantoxin-III) [37] from the same species. It could be useful to explore whether ω-Buthitoxin-Hf1a contains a common motif that is important for modulating voltage-gated ion channels in future studies. Synthetic ω-Buthitoxin-Hf1a was prepared as both C-terminally amidated (Hf1a-NH_2_) and free C-terminus (Hf1a-OH) versions. It is worth mentioning that venom peptides have been widely explored for insecticidal potential with certain success achieved by sodium channels targeting spider and scorpion toxins [38,39,40]. We also evaluated the insecticidal activities of ω-Buthitoxin-Hf1a against sheep blowflies (see results and methods in Supplementary Materials). However, in our assay, neither Hf1a-OH nor Hf1a-NH_2_ showed observable insecticidal activity against adult blowflies at doses of up to 10 μg/fly.

The NMR structure was determined for the synthetic C-terminally amidated form, and the peptide was surprisingly found to be highly structured, comprising two β-turns. N-terminal His and C-terminal Trp are, in contrast, disordered in the structure, and a lack of NOE contacts to their side chains suggests that they are flexible in solution. The structure is predominantly stabilized by the disulfide bond, but also by the experimentally verified backbone hydrogen bonds 6HN-3O and 9HN-6O. There are potentially additional side chain hydrogen bonds involving Thr3, Ser6, and His8. Although interactions involving these residues are observed in some structural models, fast chemical exchange between the hydroxyl protons and the solvent prevented experimental verification. Neither Hf1a-NH_2_ nor Hf1a-OH showed activity against L- and N-type HVA Ca_V_s, indicating a preference for T-type Ca_V_s. Further electrophysiological characterization revealed that Hf1a-NH_2_ is the active form, which showed concentration-dependent partial inhibition of Ca_V_3.2 (IC_50_ = 1.18 ± 0.94 μM, *n* = 4) and Ca_V_3.3 (IC_50_ = 0.49 ± 0.24 μM, *n* = 4) whole-cell currents but not Ca_V_3.1. Interestingly, our molecular docking results also revealed a Hf1a-NH_2_ docked in the central pore region of Ca_V_3.2 and Ca_V_3.3 but not in Ca_V_3.1. Although Ca_V_3.1, Ca_V_3.2 and Ca_V_3.3 residues near the selectivity filter are mostly conserved, minor differences may influence channel interactions with the toxin. The selectivity of Hf1a-NH_2_ against T-type Ca_V_3.2 and Ca_V_3.3 in vitro suggested potential in pain treatment, and not surprisingly, it showed promising analgesic effects in vivo in a mouse model of acute post-surgical pain. Intraplantar administration of 20 pmol/paw (1 μM; 20 μL) Hf1a-NH_2_ significantly reversed thermal allodynia in 24 h post-surgery mice compared to vehicle-treated mice, which is a comparatively low effective dose for a 10aa small peptide and close to the IC_50_s of Ca_V_3.2 and Ca_V_3.3 inhibition. The good antiallodynic potency of Hf1a-NH_2_ for thermal pain indicates its favorable therapeutic potential to relieve peripheral sensitization. Additionally, the intraplantar administration of 0.1 and 0.2 nmol/paw (5 μM and 10 μM; 20 μL) Hf1a-NH_2_ significantly reversed incision-induced mechanical allodynia in 24 h post-surgery mice, which further confirmed its promising therapeutic potential in the treatment of post-surgical pain.

## 4. Materials and Methods

### 4.1. Cell Culture

The human embryonic kidney 293 (HEK 293) cell lines (from Emmanuel Bourinet, Montpellier, France) stably expressing Ca_V_3.2 or Ca_V_3.3 [41] were cultured under 5% carbon dioxide at 37 °C in Dulbecco’s Modified Eagle Medium (DMEM) Glutamax (Gibco, Life Technologies, Carlsbad, CA, USA) supplemented with 10% (*v*/*v*) fetal bovine serum (FBS), 100 U/mL penicillin, 100 μg/mL streptomycin (Gibco, Life Technologies), and 750 μg/mL geneticin (G418) (Gibco, Life Technologies). The Chinese Hamster Ovary (CHO) cell lines (Emmanuel Bourinet, Montpellier, France) expressing Ca_V_3.1 [42] were cultured under 5% carbon dioxide at 37 °C in Alpha Minimum Essential Media (MEM) Glutamax (Gibco, Life Technologies), supplemented with 10% (*v*/*v*) fetal bovine serum (FBS) and 300 μg/mL geneticin (G418) (Gibco, Life Technologies). The human neuroblastoma SH-SY5Y cells (Victor Diaz, Goettingen, Germany) were cultured under 5% carbon dioxide at 37 °C in RPMI 1640 antibiotic-free medium (Invitrogen, Carlsbad, CA, USA), supplemented with 15% FBS and 2 mM GlutaMAX™ (Invitrogen). D-PBS (Gibco, Life Technologies) was used to wash the cells, and 0.25% Trypsin-EDTA (Gibco, Life Technologies) was used to detach the cells from the flask surface. They were split in a ratio of 1:5 (ideally 1000 cells/cm^2^) when they reached 70–80% confluence (every 2–3 days).

### 4.2. T-Type Calcium Channel Window Current FLIPR Assays

HEK 293 cells stably expressing Ca_V_3.2 were seeded into 384-well black wall clear bottom plates (Corning, Lowell, MA, USA) at a density of 30,000 cells per well. Once the cells reached 90–95% confluence after 24 h, the media were removed from the wells and replaced with 20 μL of 10% calcium 4 dye (Molecular Devices, Sunnyvale, CA, USA) in HBSS-HEPES (containing 5 mM KCl, 10 mM HEPES, 140 mM NaCl, 10 mM glucose, and 0.5 mM CaCl_2_, pH 7.4) with 0.1% bovine serum albumin (BSA). The cells were incubated for 30 min at 37 °C in the presence of 5% carbon dioxide. Each well on the reagent plates for the first addition was loaded with 15 µL fractionated venom or crude venom and incubated for 20 min after loading. The positive and negative controls contained 15 μL of HBSS-HEPES (0.1% BSA) alone. The plates were placed in the FLIPR^TETRA^ (Molecular Devices, Sunnyvale, CA, USA) programed to measure maximum fluorescence intensity following a second addition of the agonist 5 mM CaCl_2_. The fluorescence readings were recorded and converted as described previously [43], and HBSS-HEPES (0.1% BSA) was used in the second addition as a negative control. Pimozide (Ca_V_3.2, IC_50_ = 4.2 ± 1.06 μM (*n* = 3); Ca_V_3.3, IC_50_ = 7.71 ± 0.70 μM (*n* = 3)) and mibefradil (Ca_V_3.2, IC_50_ = 1.02 ± 0.14 μM (*n* = 3); Ca_V_3.3, 2.0 ± 0.2 μM (*n* = 3)) have been used as control molecules, and the achieved IC_50_s are consistent with the literature [31].

### 4.3. Crude Venom Collection and Preparation for Screening

Crude venoms of scorpions were collected by aggravation and/or mild electrical stimulation of the telson. Venom samples were then lyophilized and kept frozen until use. Each dried venom sample collected was dissolved in water to achieve a final concentration of 10 mg/mL. For the cell-based FLIPR high-throughput screening assay, each venom sample was further diluted in HBSS-HEPES with 0.1% bovine serum albumin (BSA) to prepare three venom samples, each weighing 10 µg.

### 4.4. Crude Venom HPLC Fractionation

Solvent A consisted of 0.05% trifluoroacetic acid (TFA) in milli-Q water, while solvent B consisted of 0.043% TFA and 90% acetonitrile (ACN) in water. One milligram of lyophilized crude venom dissolved in water with 5% solvent B were loaded using an UltiMate 3000 analytical autosampler (Dionex, Sunnyvale, CA, USA) onto a 00G-4053-E0 *Jupiter*^®^ (Phenomenex, Torrance, CA, USA) 5 µm *C18* 300 Å, 250 × 4.6 mm analytical reversed phase high performance liquid chromatography (RP-HPLC) column and eluted at a flow rate of 0.7 mL/min over 75 min. Elution was monitored at 214 nm.

The following gradient generated by an UltiMate 3000 pump was used to fractionate the crude venoms: A constant 5% solvent B over 5 min, 5–20% solvent B over 5 min, 20–40% solvent B over 40 min, 40–80% solvent B over 5 min, a constant 80% solvent B over 2 min, 80–5% solvent B over 3 min, and a constant 5% solvent B over 15 min.

A solvent blank run using the same gradient and equilibration with 5% solvent B for 15 min preceded each separation. Fractions were collected every 30 s or 60 s over 75 min with a Gilson FC 204 automatic fraction collector (Gilson, Middleton, WI, USA). Collected fractions were transferred into 1.5 mL Eppendorf tubes, dried in a speed vacuum concentrator, resuspended in 100 µL of milli-Q water, vortexed, and stored at −20 °C prior to assaying. All solvents used were HPLC grade.

### 4.5. Mass Spectrometry Analysis

#### 4.5.1. Matrix-Assisted Laser Desorption/Ionization Time-of-Flight Mass Spectrometry

Venom peptide molecular masses were verified by matrix-assisted laser desorption/ionization time-of-flight mass spectrometry (MALDI-TOF MS) using a 4700 Proteomics Bioanalyzer (Applied Biosystems, Foster City, CA, USA). MALDI matrices are highly purified and recrystallized reagents. Venom fractions in water obtained from RP-HPLC were mixed with the matrix α-Cyano-4-hydroxycinnamic acid (CHCA) (5 mg/mL in 50% ACN, 1% formic acid) in a 1:1 (*v*/*v*) ratio and spotted on a MALDI plate. MALDI-TOF spectra were collected in reflector positive mode, and the reported values are the average [M + H]^+^ masses.

#### 4.5.2. Electrospray Ionization Mass Spectrometry

The API2000 LC-MS/MS system (Applied Biosystems, Foster City, CA, USA) was used to further analyze the molecular weight of synthetic Hf1a-NH_2_ through electrospray ionization mass spectrometry (ESI MS). The sample was dissolved with 15% ACN in 85% milliQ water and chromatographically separated after loaded (0.2 μL) at a flow rate of 0.2 mL/min using solvent B of 50% methanol in water with nebulizing gas flow at 1.5 L/min. Mass data were then acquired via measurement of ion intensity.

### 4.6. N-Terminal Sequencing

Pure peptides (>90%) for N-terminal sequencing were prepared from RP-HPLC and dried using a freeze dryer. Peptide concentrations were measured using NanoDrop One (Thermo Scientific, Waltham, MA, USA). The lyophilized sample was then reconstituted in 0.1% TFA/20% ACN and the N-terminal sequencing was conducted by an Australian Proteome Analysis Facility Ltd. (APAF, Sydney, Australia).

### 4.7. Peptide Synthesis

The synthesis of Hf1a was performed on a Symphony automated peptide synthesizer (Gyros Protein Technologies, Inc., Tucson, AZ, USA), using Fmoc (N-(9-fluorenyl)methoxycarbonyl) protocols. Piperidine (30%) in N,N-dimethylformamide (DMF) was used for Fmoc de-protection (1 × 1.5 min, then 1 × 3 min). Fmoc aa (200 mM in DMF; 2.5 mL) was activated using 2-(1*H*-benzotriazol-1-yl)-1,1,3,3-tetramethyluronium hexafluorophosphate (HBTU; 200 mM in DMF; 2.5 mL) and diisopropylethylamine (DIPEA; 7.5% in DMF; 1.25 mL) for coupling reactions (2 × 20 min). Fmoc deprotection and coupling reactions for peptide elongation were repeated for each aa. Peptides were released from the Rink amide resin (0.176 g, 0.25 mmol; for peptide amides) or 2-Chlorotrityl chloride resin (0.176 g, 0.25 mmol; for peptide acids) and simultaneously deprotected, precipitated with ice-cold diethyl ether, and HPLC purified. The linear peptide was folded in an oxidation buffer of pH 8 containing a mix of oxidized (GSSG 0.3 mM) and reduced glutathione (GSH 0.15 Mm) for 24 h and then HPLC purified to obtain the final folded peptide products. The synthetic peptides were analyzed in MALDI-TOF MS and ESI MS to confirm matching masses with the native peptides, analyzed for purity in HPLC, and subjected to isocratic (16% solvent B) HPLC analysis for co-elution with the native peptides, monitored at 214 nm.

### 4.8. NMR Spectroscopy

An NMR sample of Hf1a was prepared by dissolving 1 mg of peptide in a 500 μL solution of H_2_O/D_2_O (90:10 *v*/*v*), at pH ~3.5. ^1^H one-dimensional data as well as ^1^H-^1^H two-dimensional TOCSY (Total Correlation Spectroscopy) [44], with a mixing time of 80 ms, and NOESY (Nuclear Overhauser Spectroscopy) [45] with a mixing time of 300 ms, experiments were recorded at 288 K on a 700 MHz Bruker Avance III HD spectrometer equipped with a cryoprobe. TOCSY experiments were recorded with 8 scans for 512 increments, while NOESY experiments were recorded with 40 scans and 512 increments. Both TOCSY and NOESY experiments were recorded with a sweep width of 12 ppm. ^1^H-^13^C as well as ^1^H-^15^N HSQC (Heteronuclear Single Quantum Coherence) spectra were also recorded at natural abundance. The ^13^C HSQC experiment was recorded with 128 scans and 256 increments, with a sweep width of 12 ppm in the F2 dimension and 80 ppm in the F1 dimension. The ^15^N HSQC experiment was recorded with 256 scans and 128 increments, with a sweep width of 12 ppm in the F2 dimension and 32 ppm in the F1 dimension. The data were subsequently processed using Topspin 4.0.3 (Bruker, Billerica, MA, USA), with a solvent signal reference of 4.77 ppm at 298 K. Sequential assignment strategies [46] were used to assign and analyze the data in the program CARA (Computer Assisted Resonance Assignment) [47]. Additional TOCSY data at varying temperatures (280 K, 293 K, 298 K, 303 K) were recorded to monitor the temperature dependence of the amide protons.

### 4.9. Structure Calculations

Structure determination was performed using established procedures [48]. Structure calculations were conducted using restraints generated from multiple sources. Inter-proton distance restraints were generated from the peak volumes of the cross peaks present in the NOESY spectra. Torsion Angle Likelihood Obtained from Shift and sequence similarity (TALOS-N) [49] was used to predict dihedral ϕ (C_−1_-N-Cα-C) and ψ (N-Cα-C-N_+1_) backbone angles. Hydrogen bonds were identified via a combination of preliminary structure calculations and backbone amide temperature coefficients. The chemical shift of the HN proton of each residue was plotted against temperature, with values > −4.6 ppb/K for the coefficient of the linear relationship being taken as indicative of a hydrogen bond being donated by that HN [50]. Hydrogen bond acceptors were identified through preliminary structural calculations. Initial structures were calculated using the program CYANA (Combined Assignment and dYnamics Algorithm for NMR Applications) [51] using an automated NOESY assignment. Final validated distance restraints, dihedral restraints, and hydrogen bond restraints were all used as input for the program CNS (Crystallography and NMR System). Simulated annealing was performed by CNS to generate 50 structures [52]. The generated structures were subsequently water minimized using Cartesian dynamics to generate the final structures for Hf1a. Stereochemical analysis was conducted using MolProbity [53] by comparing the generated structures to those of previously published structures in the RCSB. The program MOLMOL [54] was used to display and produce images of the secondary and tertiary structures of the best 20 structures. These structures have no violations of distances or dihedral angles above 0.2 Å or 2°, and have low energy and excellent stereochemistry.

### 4.10. Whole-Cell Patch-Clamp Electrophysiology

Whole-cell patch-clamp experiments were performed on an automated electrophysiology platform QPatch 16 X (Sophion Bioscience A/S, Ballerup, Denmark) in a single-hole configuration using 16-channel planar patch chip QPlates (Sophion Bioscience A/S). The extracellular recording solution contained, in mM: TEACl 157, MgCl_2_ 0.5, CaCl_2_ 5, and HEPES 10; pH 7.4 adjusted with TEAOH; and osmolarity 320 mOsm. The intracellular pipette solution contained, in mM: CsF 140, EGTA 1, HEPES 10, and NaCl 10; pH 7.2 adjusted with CsOH; and osmolarity 325 mOsm. Compounds were diluted in an extracellular recording solution with 0.1% BSA at the concentrations stated (DMSO ≤ 0.1%), and the effects of compounds were compared to the control (extracellular solution with 0.1% BSA) parameters within the same cell. Compounds’ incubation time varied from two (for the highest concentration) to five (for the lowest concentration) minutes by applying the voltage protocol 10–30 times at 10 s intervals to ensure that steady-state inhibition was achieved. The effects of the compounds were obtained using 200 ms voltage steps to peak potential from a holding potential of −90 mV. Current–voltage (*I*–*V*) relationships were obtained by holding the cells at a potential of −100 mV before applying 50 ms pulses to potentials from −75 to +45 mV every 5 s in 5 mV increments. Data were fitted with a single Boltzmann distribution: *I*/*I*_max_ = {1 + exp[*V* − *V*_5_/*k*}^−1^, where *V*_50_ is the half-availability voltage and *k* is the slope factor. Off-line data analysis was performed using QPatch Assay Software v5.6 (Sophion Bioscience A/S) and Excel 2013 (Microsoft Corporation, Redmond, WA, USA).

### 4.11. HVA Calcium Channel FLIPR Assays

SH-SY5Y cells were seeded into 384-well black wall clear bottom plates at a density of 15,000 cells per well, resulting in 90–95% confluence after 24 h. The media were then removed from the wells and replaced with 20 μL of 10% calcium 4 dye (Molecular Devices) in physiological salt solution (PSS) (containing 5.9 mM KCl, 1.4 mM MgCl_2_, 10 mM HEPES, 1.2 mM NaH_2_ PO_4_, 5 mM NaHCO_3_, 140 mM NaCl, 11.5 mM glucose, and 1.8 mM CaCl_2_, pH 7.4) with 0.1% BSA. As reported [55], for N-type calcium channel FLIPR assays, the cells were pre-incubated with 10 µM nifedipine added to the dye to ensure full inhibition of L-type calcium responses. For L-type calcium channel FLIPR assays, the cells were pre-incubated with 1 µM CVID added to the dye to ensure full inhibition of N-type calcium responses. The positive control on the first reagent plate contained 15 μL of PSS (0.1% BSA), whereas PSS (0.1% BSA) containing 1 µM CVID and 10 µM nifedipine (final concentration) was used as a negative control. The fluorescence readings were recorded and converted as described previously [55], and an agonist containing 90 mM KCl + 5 mM CaCl_2_ was used in the second addition.

### 4.12. Molecular Docking

The molecular docking of Hf1a was performed by using previously published Cryo-EM structures of human Ca_V_3.1 (PDB 6KZO) [56] and Ca_V_3.3 (PDB 7WLI) [34] and the Ca_V_3.2 model (Uniprot sequence code: O95180) built from the AlphaFold 2 protein structure database [57]. The resulting model of Ca_V_3.2 was energy minimized using the GROMOS force field and then validated by Ramachandran plot analysis. The structures of Ca_V_3.1, Ca_V_3.2, and Ca_V_3.3 were visualized in PyMol, and molecular docking was performed using Autodock Vina [58]. To define the search space, the grid box with the following dimensions was used: for Ca_V_3.1, center x = 175.853, center y = 167.509, center z = 197.125, for Ca_V_3.2, center x = 140.956, center y = 128.702, center z = 145.705, and for Ca_V_3.3 center x = 138.563, center y = 127.235, center z = 147.072. The size of the grid box for all the docking in T-type Ca_V_s was as follows: size x = 20, size y = 20, size z = 20. The exhaustiveness of the search was set to 8.0.

### 4.13. Animals

All behavioral experiments were performed with adult male C57BL/6J mice (age 6–8 weeks; weight 19–23 g), housed under 12 h light-dark cycles, in groups of 5 per cage with access to water and standard rodent chow ad libitum. The in vivo experiments in mice were conducted in accordance with the Guide for the Care and Use of Laboratory Animals and the International Association for the Study of Pain Guidelines for the Use of Animals in Research. Ethical approval for experiments involving animals was obtained from the Institutional Animal Care and Use Committee of Jiangsu University (protocol code 11746). All measurements were performed at room temperature and by a blinded observer unaware of the treatment that each animal received. The general health and plantar foot well-being of the mice were monitored daily, and the sample sizes of each experiment are detailed in the corresponding figure legends.

### 4.14. Surgery

Surgery of mice was performed as previously described, with minor adjustments [35]. Mice were anesthetized by inhalation with 2.5% isoflurane for induction, followed by 1% isoflurane for maintained anesthesia via a nose cone. A longitudinal incision of 7 mm was made on the plantar (glabrous) surface of the right hind paw through the skin and fascia using a number 11 sterile surgical scalpel after disinfection with 70% ethanol. The incision started about 3 mm from the heel side and extended toward the toes. The underlying *flexor digitorum brevis* muscle was elevated with sterile forceps to mimic muscle retraction and was incised longitudinally with the origin and insertion intact. Gentle pressure was applied to the wound with sterile gauze to stop bleeding, followed by wound closure with two sterile silk sutures using the simple interrupted suture technique. A 5% povidone-iodine solution was spread over the closed wound, and inhalation anesthesia was stopped. The mice were then moved back to their cages for recovery and observed for abnormal behaviors, including signs of spontaneous pain and paw guarding, for 10 min. Sham surgeries involved identical handling of mice, including anesthesia and disinfections, and two sterile silk sutures were stitched into the skin without any incisions made.

### 4.15. Mechanical Paw Withdrawal Threshold Measurements

Mechanical paw withdrawal thresholds were assessed 24 h post-surgery using the Ugo Basile Electronic von Frey apparatus (38450, Ugo Basile, Varese, Italy) following a previously described method [35]. Briefly, the animals were habituated in individual enclosures for at least 30 min before the measurements. Using a soft-tipped von Frey filament, the pressure against the glabrous plantar surface (adjacent to the wound) of the right hind paw was slowly increased through rotation of the device handle, with the force increasing at a rate of 1 g/s, and the force (g) causing paw withdrawal (lift, shake, lick) was displayed by the apparatus. One biological replicate was averaged from three measurements separated by 5-min intervals.

### 4.16. Thermal Paw Withdrawal Latency Time Measurements

Thermal paw withdrawal latency time was assessed 24 h post-surgery using the Ugo Basile Plantar Test apparatus (37370, Ugo Basile, Varese, Italy) following the Hargreaves radiant heat method [59]. Mice were placed in individual mouse runs at least 30 min prior to testing to acclimatize to the room conditions. An infrared radiant heat source (intensity set as 60) under a heat-acrylic plastic floor was focused on the surgical paw. The time to withdrawal was automatically measured, and the cut-off time was set as 20 s to avoid thermal injury. One biological replicate was averaged from three measurements separated by 5-min intervals.

### 4.17. Locomotor Performance Assessment

The locomotor effects of intraplantar administration of Hf1a_NH_2_ and MVIIA (yuanpeptide Co., Ltd., Nanjing, China) were measured using a Process Control Treadmill apparatus (TSE Systems, Berlin, Germany) by assessing the distance covered and motor function of the mice. The distance traveled (m) were assessed for 1 min 24 h post-injection of the peptides with the initial speed set as 0.1 m/s and was automatically increased by 0.14 m/s at the end of each measurement (speed of 0.15 m/s suggested for healthy mice [60] was noticed to be unachievable for post-surgery mice in the current study). A 20 s training at a constant speed of 0.1 m/s was set for each mouse before the formal measurements. The distance traveled (m) was automatically recorded by the instrument, and abnormal motor function of the mice could be observed from top of the treadmill.

### 4.18. Treatments

Hf1a_NH_2_ doses of 1.1 μg/kg (1 μM), 5.7 μg/kg (5 μM), and 11 μg/kg (10 μM) were prepared on the day of the experiment in phosphate-buffered saline (PBS; 1X, Cytiva HyClone), and MVIIA (0.8 μg/kg; 300 nM) was also prepared on the day of the experiment in PBS as a control peptide. Local effects of Hf1a_NH_2_ and MVIIA were administered by intraplantar injection into the right hind paw in a volume of 20 µL/paw using a sterile 30 G needle 24 h after surgery, and their effects were compared to the vehicle (PBS) control. Mechanical and thermal allodynia were measured 20 min following the intraplantar administration of Hf1a_NH_2_ and MVIIA, and locomotor activities were measured 24 h after the administration of Hf1a_NH_2_ and MVIIA.

### 4.19. Data Analysis

The data were plotted and analyzed using GraphPad Prism v8.0 (GraphPad Software Inc., San Diego, CA, USA). A four-parameter logistic Hill equation with variable Hill coefficients was fitted to the data for the concentration-response curves. Data are means ± SEM of *n* independent experiments. Statistical analysis was performed using one-way ANOVA with Dunnett’s multiple comparisons test, with statistical significance at *p* < 0.05.

## 5. Conclusions

In summary, assay-guided fractionation of scorpion *H. franzwerneri* crude venom led to the discovery of novel Ca_V_3.2 and Ca_V_3.3 inhibitory peptide ω-Buthitoxin-Hf1a. The synthetic peptide with C-terminal amidation (Hf1a-NH_2_) showed selective and potent partial inhibition of Ca_V_3.2 and Ca_V_3.3 whole-cell currents in vitro and induced promising analgesic effects on both mechanical and thermal allodynia in vivo in a mouse model of acute post-surgical pain.

## Data Availability

The NMR structure and chemical shift assignments for Hf1a were submitted to PDB and BMRB and given accession codes 9BFL and 31169, respectively.

## Supplementary Materials

The supporting information can be downloaded at: https://www.mdpi.com/article/10.3390/ijms25094745/s1.
