# Supplementary material for: Novel Scorpion Toxin ω-Buthitoxin-Hf1a Selectively Inhibits Calcium Influx via CaV3.3 and CaV3.2 and Alleviates Allodynia in a Mouse Model of Acute Postsurgical Pain
Source: Int J Mol Sci. 2024 Apr 26;25(9):4745. doi: 10.3390/ijms25094745 (PMC11084959; doi:10.3390/ijms25094745)
Supplement: Supplementary file 1 [file ijms-25-04745-s001.zip › ijms-2948123-supplementary.pdf]

SUPPORTING INFORMATION

Novel Scorpion Toxin ω-Buthitoxin-Hf1a Selectively Inhibits Calcium Influx via Cav3.3 and Cav3.2 and Alleviates Allodynia in a Mouse Model of Acute Postsurgical Pain

Dan Wang [1,2,*], Volker Herzig [3,4], Zoltan Dekan [2], K. Johan Rosengren [5], Colton D. Payne [5], Md. Mahadhi Hasan [6], Jiajie Zhuang [1], Emmanuel Bourinet [7], Lotten Ragnarsson [2], Paul F. Alewood [2] and Richard J. Lewis [2,*]

[1] Department of Chinese Medicine and Pharmacy, School of Pharmacy, Jiangsu University,
Zhenjiang 212013, China; 3201603097@stmail.ujs.edu.cn
[2] Division of Chemistry and Structural Biology, Institute for Molecular Bioscience, The University of
Queensland, Brisbane, QLD 4072, Australia; ivydan92@gmail.com (Z.D.); l.ragnarsson@imb.uq.edu.au (L.R.); p.alewood@imb.uq.edu.au (P.F.A.)
[3] Centre for Bioinnovation, University of the Sunshine Coast, Sippy Downs, QLD 4556, Australia; vherzig@usc.edu.au
[4] School of Science, Technology and Engineering, University of the Sunshine Coast, Sippy Downs, QLD 4556, Australia
[5] School of Biomedical Sciences, The University of Queensland, Brisbane, QLD 4072, Australia; j.rosengren@uq.edu.au (K.J.R.); c.payne@uq.net.au (C.D.P.)
[6] Pharmacy Discipline, Life Science School, Khulna University, Khulna 9208, Bangladesh; mahadhi@pharm.ku.ac.bd
[7] Institute of Functional Genomics, Montpellier University, CNRS, INSERM, 34090 Montpellier, France; emmanuel.bourinet@igf.cnrs.fr
* Correspondence: danwang@ujs.edu.cn (D.W.); r.lewis@imb.uq.edu.au (R.J.L.); Tel.: +61-7-3346-2984 (R.J.L.)

1. Supplementary Results

1.1. High-throughput FLIPR venom screening at Cav3.2

55 crude venoms from 52 scorpion species of 20 genera across 6 families of scorpions were selected from the collected venom library, and screened at Cav3.2 FLIPR window current assays with 10 μg/well (Figure S1). Inhibitory crude venoms with >50% inhibition are presented in Table S1.

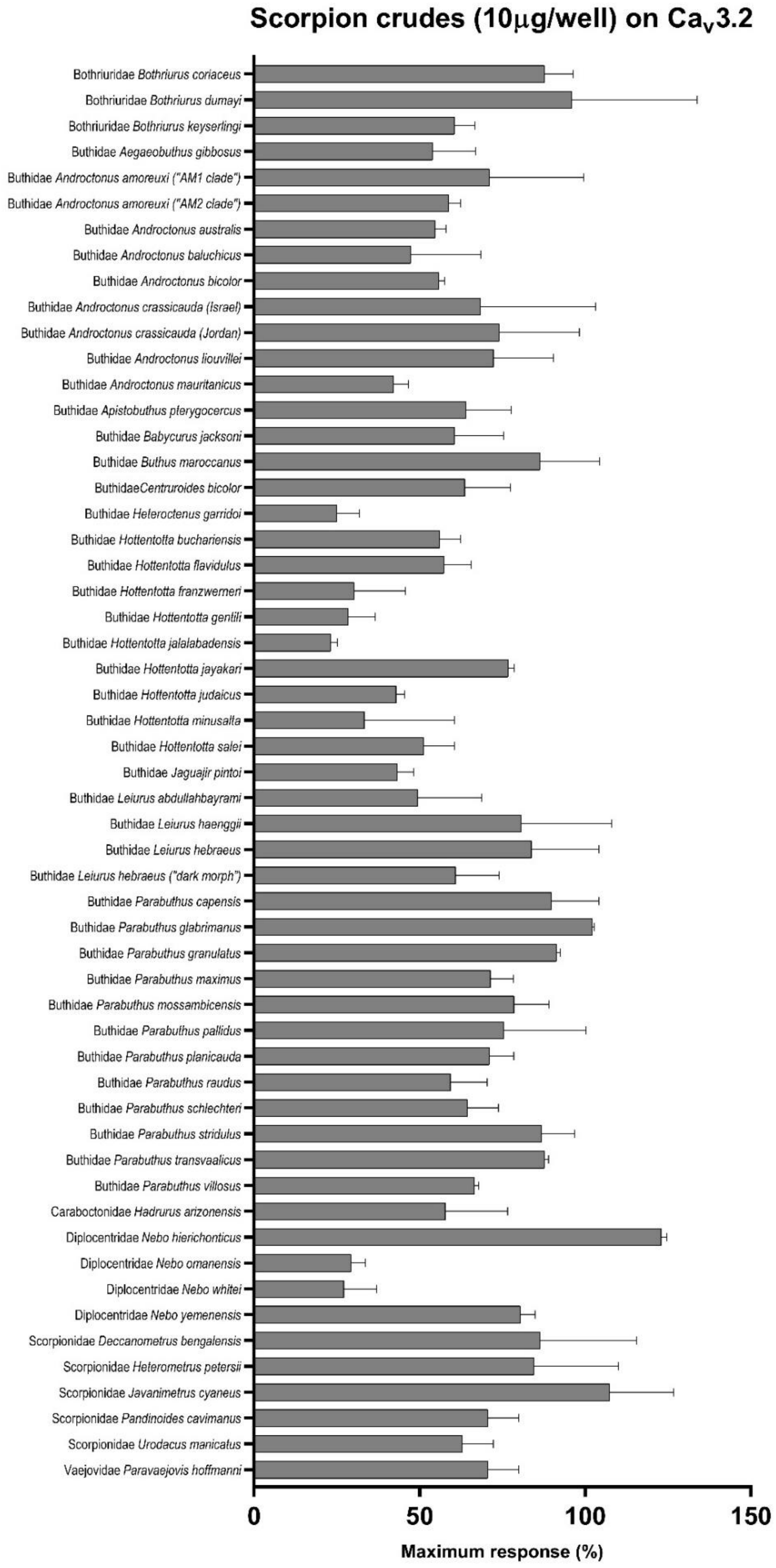

Figure S1. Activity of 55 scorpion crude venoms at Cav3.2 expressed in HEK293 cells. Data are means ± SEM of single experiments per venom performed in duplicate.

Table S1. Crude venoms with >50% inhibition of Cav3.2 FLIPR responses

Inhibition	Genus	Species	Sex	Family	Organism
>70%	*Hottentotta*	*franzwerneri*	m	Buthidae	scorpion
>70%	*Hottentotta*	*gentili*	m	Buthidae	scorpion
>70%	*Hottentotta*	*jalalabadensis*	f	Buthidae	scorpion
>70%	*Heteroctenus*	*garridoi*	m	Buthidae	scorpion
>70%	*Nebo*	*omanensis*	f	Diplocentridae	scorpion
>70%	*Nebo*	*whitei*	f	Diplocentridae	scorpion
>60%	*Hottentotta*	*minusalta*	f	Buthidae	scorpion
>50%	*Androctonus*	*baluchicus*	m	Buthidae	scorpion
>50%	*Androctonus*	*mauritanicus*	n/a	Buthidae	scorpion
>50%	*Jaguajir*	*pintoi*	n/a	Buthidae	scorpion

1.2. Determination of peptide mass and NMR structure

Figure S2 shows the main results in MALDI TOF MS of scorpion *Hottentotta franzwerneri* fractions I (1195.4 Da) and II (1196.4 Da) in positive ion mode. After the purity (>98%) of synthetic Hf1a-NH_2 being confirmed by HPLC and correct mass confirmed by electrospray ionization mass spectrometry (ESI MS) (Figure S3), its NMR statistics data was acquired and summarized in Table S2.

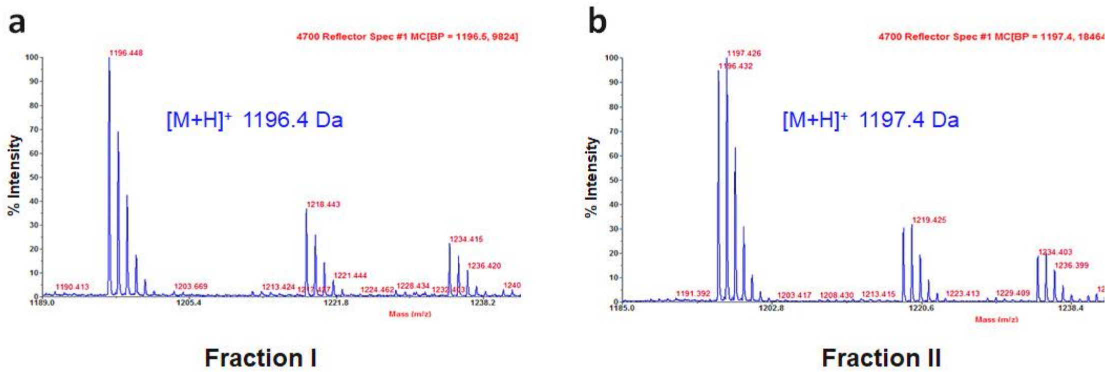

Figure S2. (**a**) MALDI TOF MS result of scorpion *H. franzwerneri* fraction I with a main average mass of 1195.4 Da. (**b**) MALDI TOF MS result of scorpion *H. franzwerneri* fraction II with a main average mass of 1196.4 Da.

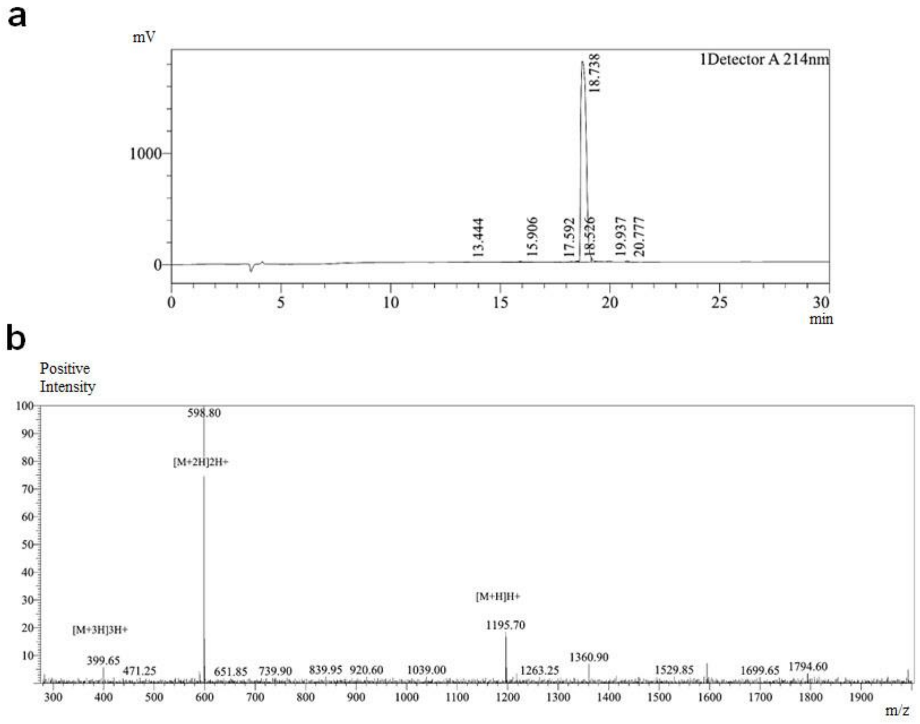

Figure S3. (**a**) RP-HPLC trace of synthetic Hf1a-NH_2 using the gradient of 5–35% solvent B over 30 min, where Solvent A consists of 0.1% trifluoroacetic acid (TFA) in milli-Q water, while solvent B consists of 0.1% TFA in acetonitrile. (**b**) ESI MS result of synthetic Hf1a-NH_2 with observed mass of 1195.6 Da.

Table S2. NMR statistics data for Hf1a-NH_2

**Energies (kcal/mol)**	**Hf1a-NH$_2$**
Overall	-275.67 ± 12.79
Bonds	3.84 ± 0.45
Angles	9.31 ± 1.42
Improper	3.15 ± 1.18
Dihedral	43.31 ± 1.01
Van der Waals	-27.21 ± 4.17
Electrostatic	-308.23 ± 11.80
NOE	0.033 ± 0.010
Cdih	0.118 ± 0.134
**MolProbity Statistics**	
Clashes (>0.4 Å / 1000 atoms)	2.13 ± 3.13
Poor rotamers	0.05 ± 0.22
Ramachandran Outliers (%)	0
Ramachandran Favoured (%)	100

MolProbity score	0.87 ± 0.42
Molprobity score percentile	99.3 ± 0.92
Residues with bad bonds	0.15 ± 0.37
Residues with bad angles	0
**Atomic RMSD (Å)**	
Mean global backbone (residues 2-9)	0.43 ± 0.13
Mean global heavy (residues 2-9)	1.06 ± 0.21
**Experimental Restraints**	
**Distance restraints**	
Short range (i-j < 2)	55
Medium range (/i-j/ < 5)	10
Long range (/i-j/ > 5)	3
Hydrogen bonds	2
Total	70
**Dihedral angle restraints**	
φ	7
ψ	4
Total	11
**Violations from experimental restraints**	
Total NOE violations exceeding 0.2 Å	0
Total Dihedral violations exceeding 2.0°	0

1.3. Evaluation of the effects of ω-Buthitoxin-Hf1a on I–V curves of Cav3.2 and Cav3.3

Current-voltage (*I–V*) relationships of Cav3.2 and Cav3.3 in the presence of 1 μM Hf1a-NH_2 revealed that block was not accompanied by shifts in the *I–V* relationship (Figure S4).

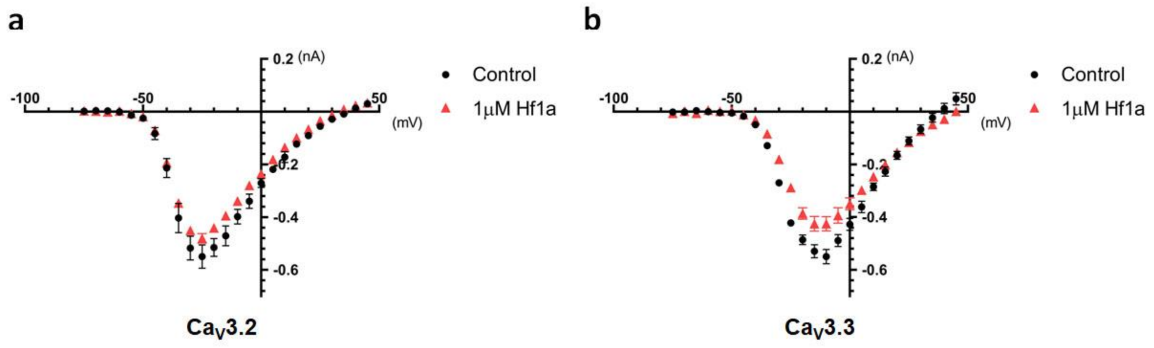

Figure S4. Comparison of the *I–V* relationships of Cav3.2 and Cav3.3 before (black) and after (red) the addition of 1 μM Hf1a-NH_2. The cells were holding at –100 mV before applying 50 ms pulses to potentials from –75 to +45 mV every 5 s in 5 mV increments. (**a**) *I–V* relationships of Cav3.2 (n = 3) plotted from –75 mV to +45 mV, V_{max} at –25 mV. (**b**) *I–V* relationships of Cav3.3 (n = 3) plotted from –75 mV to +45 mV, V_{max} at –10 mV. Error bars represent the SEM.

1.4. Locomotor Performance Assessment

Locomotor effects of 24h post administration of Hf1a-NH_2 and MVIIA were measured using a Process Control Treadmill apparatus by assessing distance covered and motor function of the mice. The distance covered for Hf1a-NH_2 and MVIIA treated mice showed no difference from vehicle treated mice, and no abnormal motor functions like foot slips were noticed during the run (Figure S5).

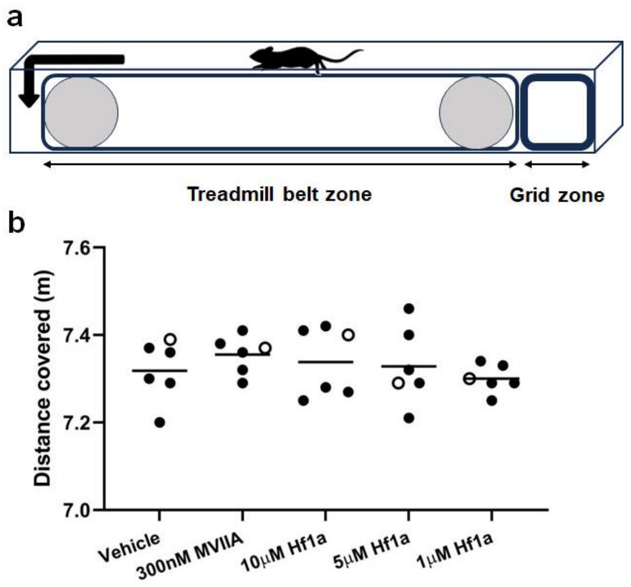

Figure S5. Locomotor behavior of mice assessed 48 h post-surgery. (**a**) Side view of the treadmill instrument; (**b**) There are no differences in the mean distance covered among Hf1a (1μM, 5μM and 10 μM Hf1a; 20 μL) injected mice, control peptide MVIIA (300 nM; 20 μL) injected mice and vehicle mice.

1.5. Evaluation of insecticidal activities of ω-Buthitoxin-Hf1a against sheep blowflies

Hf1a-NH2 and Hf1a-OH toxicity was tested against sheep blowflies (*Lucilia cuprina*), at doses1, 4 and 10 μg per fly, and no insecticidal activity was observed.

2. Supplementary Method

1.1. Evaluation of insecticidal activities against sheep blowflies

Peptide insecticidal toxicity was tested using 1, 4 and 10 μg peptide for three groups of Australian sheep blowflies (*Lucilia cuprina*), respectively, each group with 10 flies. Peptide was prepared in milliQ water, and a maximum volume of 2 μL was injected per fly using a 1.0 mL Terumo Insulin syringe (B-D Ultra-Fine, Terumo Medical Corporation, MD, USA) with a fixed 29-gauge needle fitted to an Arnold hand micro-applicator (Burkard Manufacturing Co. Ltd., Rickmansworth, UK). Flies were individually housed in 2 mL Eppendorf tubes and paralytic effects determined 0.5, 1, 2 and 24 h after injection. 20 flies injected with only milliQ water were set as control.
